# Reproducible isolation of bovine mammary macrophages for analysis of host pathogen interactions

**DOI:** 10.1186/s12917-024-03944-w

**Published:** 2024-03-09

**Authors:** Abbie Tomes, Nathan Archer, James Leigh

**Affiliations:** School of Veterinary Medicine and Science, University of Nottingham, Nottingham, UK

**Keywords:** Macrophage, Mammary gland, Mastitis, Inflammasome, *Streptococus uberis*, Pathogenesis, Ex vivo

## Abstract

**Background:**

Macrophages residing in milk are vital during intramammary infections. This study sought to develop a method enabling the investigation of macrophage responses to pathogens. *Streptococcus uberis* is the predominant cause of bovine mastitis UK-wide and its pathogenesis is unusual compared to other intramammary pathogens. Previous studies utilise macrophage cell lines, isolated bovine blood derived monocytes, or macrophages from raw milk through complex or inconsistent strategies such as fluorescence activated cell sorting (FACS), centrifugation and selective adherence, and CD14 antibody-microbeads. The centrifuge steps required in the initial stages often damage cells. Thus, the aim of this study was to develop a reliable, reproducible, and cost-effective method for isolating mammary macrophages from milk in a way that allows their culture, challenge with bacteria, and measurement of their response *ex-vivo*.

**Results:**

This method achieves an average yield of 1.27 × 10^7^ cells per litre of milk. Whole milk with somatic cell range of 45–65 cells/µL produced excellent yields, with efficient isolations accomplished with up to 150 cells/µL. This strategy uses milk diluted in PAE buffer to enable low-speed centrifugation steps followed by seeding on tissue-culture-treated plastic. Seeding 1,000,000 milk-extracted cells onto tissue culture plates was sufficient to obtain 50,000 macrophage. Isolated macrophage remained responsive to challenge, with the highest concentration of IL-1β measured by ELISA at 20 h after challenge with *S. uberis*. In this model, the optimal multiplicity of infection was found to be 50:1 bacteria:macrophage. No difference in IL-1β production was found between macrophages challenged with live or heat-killed *S. uberis*. Standardisation of the production of IL-1β to that obtained following macrophage stimulation with LPS allowed for comparisons between preparations.

**Conclusions:**

A cost-effective method, utilising low-speed centrifugation followed by adherence to plastic, was established to isolate bovine mammary macrophages from raw milk. This method was shown to be appropriate for bacterial challenge, therefore providing a cost-effective, *ex-vivo*, and non-invasive model of macrophage-pathogen interactions. The optimal multiplicity of infection for *S. uberis* challenge was demonstrated and a method for standardisation against LPS described which removes sample variation. This robust method enables, reproducible and reliable interrogation of critical pathogen-host interactions which occur in the mammary gland.

**Supplementary Information:**

The online version contains supplementary material available at 10.1186/s12917-024-03944-w.

## Background

Bovine milk contains a variety of immune cells including macrophages, neutrophils and lymphocytes. The predominant leukocyte are macrophages, constituting 10–80% of the total cell population in a healthy mammary gland [1]. Macrophages within the mammary gland can reside between epithelial cells in the mammary tissue, called ductal macrophages, or in the lumen within milk, known as bovine mammary macrophages (BMMOs) [2, 3]. BMMOs are important cells during intramammary infection as they function in both the innate and adaptive immune responses. In conjunction with epithelial cells, macrophages can act innately through the phagocytosis of invading pathogens and secretion of pro-inflammatory mediators. Additionally, macrophages play a role in the adaptive immune response by acting as antigen presenting cells to lymphocytes, including T-cells [1, 3–5].

Intramammary bacterial infection is considered to be the main cause of bovine mastitis with more than 135 bacterial species identified as causative agents [6, 7]. *Streptococcus uberis* is the predominant cause of bovine mastitis in the UK [8, 9]. Previous studies have outlined how *S. uberis* pathogenesis differs compared to other intramammary pathogens. Moyes et al., [10] showed the responses from mammary epithelial cells obtained during experimental intramammary infection were consistent with stimulation by activated macrophages rather than direct stimulation by bacteria. Consistent with this observation, Günther et al., [11], demonstrated in vitro that *S. uberis* failed to induce innate responses in primary mammary epithelial cells, but did induce responses from macrophages derived from blood, an observation subsequently reproduced in macrophages obtained from bovine milk [12]. *S. uberis* stimulates activation of the transcription factor, NF-κB (nuclear factor kappa B), within BMMOs, which translocates into the nucleus and upregulates transcription of immune genes, including interleukin-1 beta (IL-1β) [11]. Challenge of isolated BMMOs with *S. uberis* strain 0140J increased concentration of IL-1β after 24 h compared to no treatment [12]. Therefore, the immune response from BMMOs can be measured by determining the production of IL-1β.

Previous studies have used both the murine mammary macrophage cell line, RAW 246.7 [13], and bovine blood derived monocytes. Peripheral blood mononuclear cells (PBMCs) isolated from bovine blood samples were incubated in non-adherent Teflon bags for 7–8 days to allow monocyte differentiation into mature macrophages. Cell suspensions were then seeded into culture plates/dishes and macrophages were purified through selective adherence. As macrophages are the only adherent cells within the PBMC population, contaminating lymphocytes were washed away and the adherent macrophages remained [14–17].

Alternatively, monocytes were positively selected from isolated PBMCs using mouse anti-human CD14-coupled microbeads and magnetic activated cell sorting columns. CD14 (cluster of differentiation 14) is highly expressed on bovine monocytes and macrophages and so is commonly used to differentiate these cell populations [18, 19]. Purified monocytes were plated and differentiated into macrophages by the addition of 10 ng/mL recombinant bovine macrophage colony-stimulating factor (M-CSF) [20, 21]. Although the use of blood monocytes is an improvement in comparison to RAW 246.7 cells as it avoids the issues that arise with immortalisation of cells and is of bovine origin, these cells have not undergone differentiation into mammary specific macrophages and as a consequence, these cells may act differently [11].

This issue was overcome by extracting cells from raw bovine milk, generating an ex vivo model from the same population of cells normally present in the bovine mammary gland. Milk was diluted in either PBS, PAE buffer (PBS, acid-citrate dextrose, EDTA) or PBS/EDTA/TE buffer; centrifuged; the fat layer and supernatant discarded and the pellet washed [12, 16, 22]. Several methods were used to subsequently isolate BMMOs. These included BMMO identification through fluorescence-activated cell sorting (FACS) with mouse anti-human CD14 antibodies [18, 22], selective adherence [16] or mouse anti-human CD14 microbeads [12]. Another method isolated BMMOs from milk diluted with MGS (modified Gey’s balanced salt solution) and metrizamide (density gradient medium for centrifugation) and collected the bottom 10 mL after centrifugation in fractions of 1–3 mL by aspiration. Macrophages were identified by morphology and the yellow-reddish cytoplasm after acridine orange staining due to the high RNA content [23].

The ability to reliably and reproducibly isolate BMMOs from bovine milk would provide a non-invasive methodology to investigate bovine macrophages and specifically to analyse the interactions of this population of cells with invading pathogens. Due to the variation of the isolation methods within the literature, and in some cases their reliance on poorly described antibody reagents, the aim of this study was to develop a reliable method to reproducibly isolate BMMOs from bovine milk and demonstrate their utility by challenge with an important mammary pathogen, *S. uberis*.

## Results

### Isolation of BMMOs

**Fig. 1:**
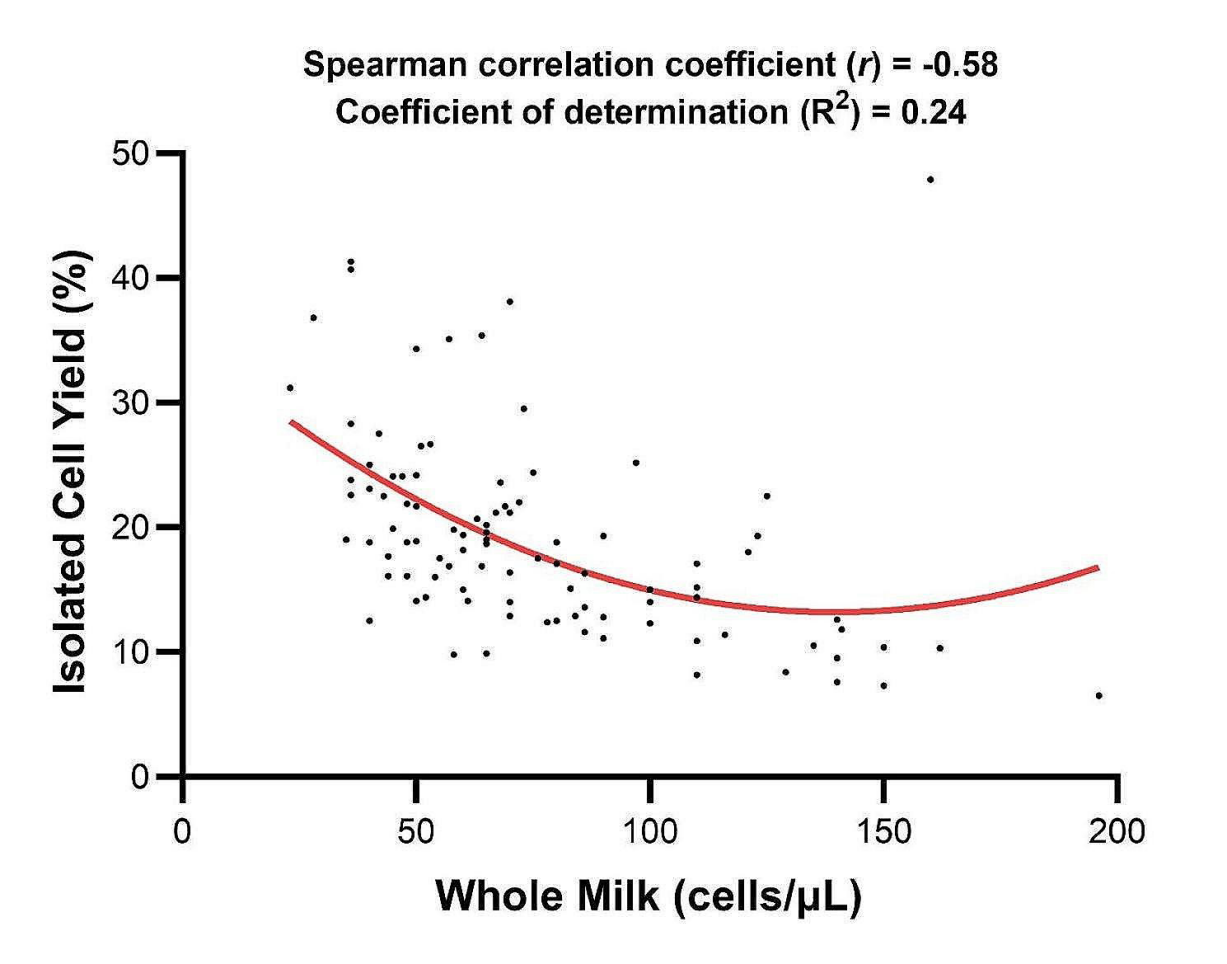
Correlation between whole milk SCC and isolated cell yield. 3 L of milk was collected from bulk tank between 2020–2023 and the somatic cell count (SCC) per µL was measured using a DeLaval cell counter. Graph was determined using data provided in Supplemental Table 1. Association between the SCCs from whole milk and isolated cell yield were determined using Spearman rank-order correlation coefficient (weak negative correlation, *r* = -0.58). Nonlinear regression calculated the curve of best fit and the coefficient of determination (R^2^ = 0.24)

The somatic cell count (SCC) of each bulk milk sample was determined prior to isolation of leukocytes and number of isolated and washed cells similarly determined to estimate the efficacy of the isolation process. Cell isolation was reliable using milk over the SCC range of 20–200 cells/µL but could be considered optimal using milk with a SCC < 100 cells/µL (Fig. 1). A weak negative correlation was found between the SCC of whole milk and the isolated cell yield (*r* = -0.58) with a weak coefficient of determination (R^2^ = 0.24). The isolation process was conducted on 96 separate 3 L bulk milk samples obtained from the University of Nottingham Dairy herd located at the Centre for Diary Science Innovation (CDSI) between September 2020–2023 (Supplemental Table 1).

**Fig. 2:**
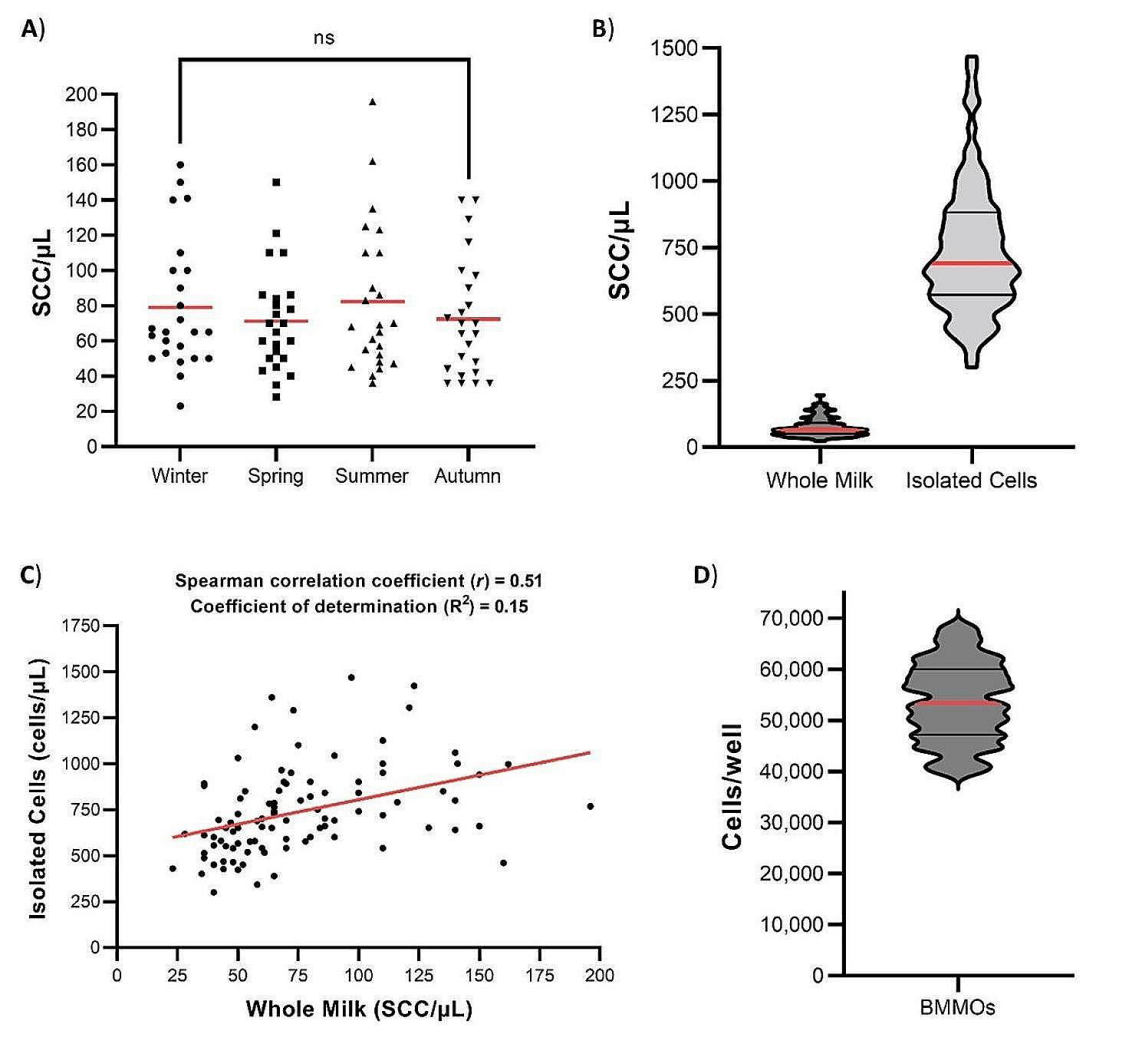
Cell numbers in milk, isolated cells and seeded BMMOs. 3 L of milk was collected from bulk tank between 2020–2023 and the somatic cell count (SCC) per µL was measured using a DeLaval cell counter. Milk was discarded if the SCC was > 200 cells/µL. Graphs were determined using data provided in Supplemental Table 1. **A**) Milk SCCs were divided seasonally and presented as mean with *N* = 24. Normality was determined by the Shapiro-Wilk test. Data were found not to be normally distributed and so was statistically analysed using a Kruskal-Wallis with Dunn’s multiple comparison post hoc test. No significant differences (ns) were found between seasons. **B**) SCCs were determined in whole milk (3 L) and following the cell isolation protocol, the isolated cell count in 50 mL PBS suspension. **C**) Association between the SCCs from whole milk and isolated cells were determined using Spearman rank-order correlation coefficient (weak positive correlation, *r* = 0.51). Nonlinear regression calculated the line of best fit and the coefficient of determination (R^2^ = 0.15). **D**) Number of isolated bovine mammary macrophages (BMMOs) were counted per well. Data (**B** and **D**) is presented as the medium, including upper and lower quartiles, with *N* = 96

The SCC of the bulk milk samples used varied from 20 to 190 cells/µL, no significant difference in the SCC with seasonality was detected (Fig. 2A). On average (*n* = 96), 3.8 × 10^7^ cells were isolated from 3 L of milk containing 2.3 × 10^8^ cells, representing an average yield of 19.1% of the total cell population. The efficiency of isolation ranged from a yield of 6.5% (from a milk sample with a SCC of 196 cells/µL) to 47.9% (from a milk sample with a SCC of 160 cells/µL) (Fig. 2B; Supplemental Table 1). A weak positive correlation was found between the SCC of whole milk and the number of isolated cells obtained (*r* = 0.51) with a weak coefficient of determination (R^2^ = 0.15) (Fig. 2C).

The isolated cells (100,000 cells) were plated onto plastic and those immobilised on plastic (BMMOs) after 18 h released to determine the actual number of BMMOs per well. Typically, plating in this manner yield an adherent population of ~ 50,000 cells per well (Fig. 2D).

**Fig. 3:**
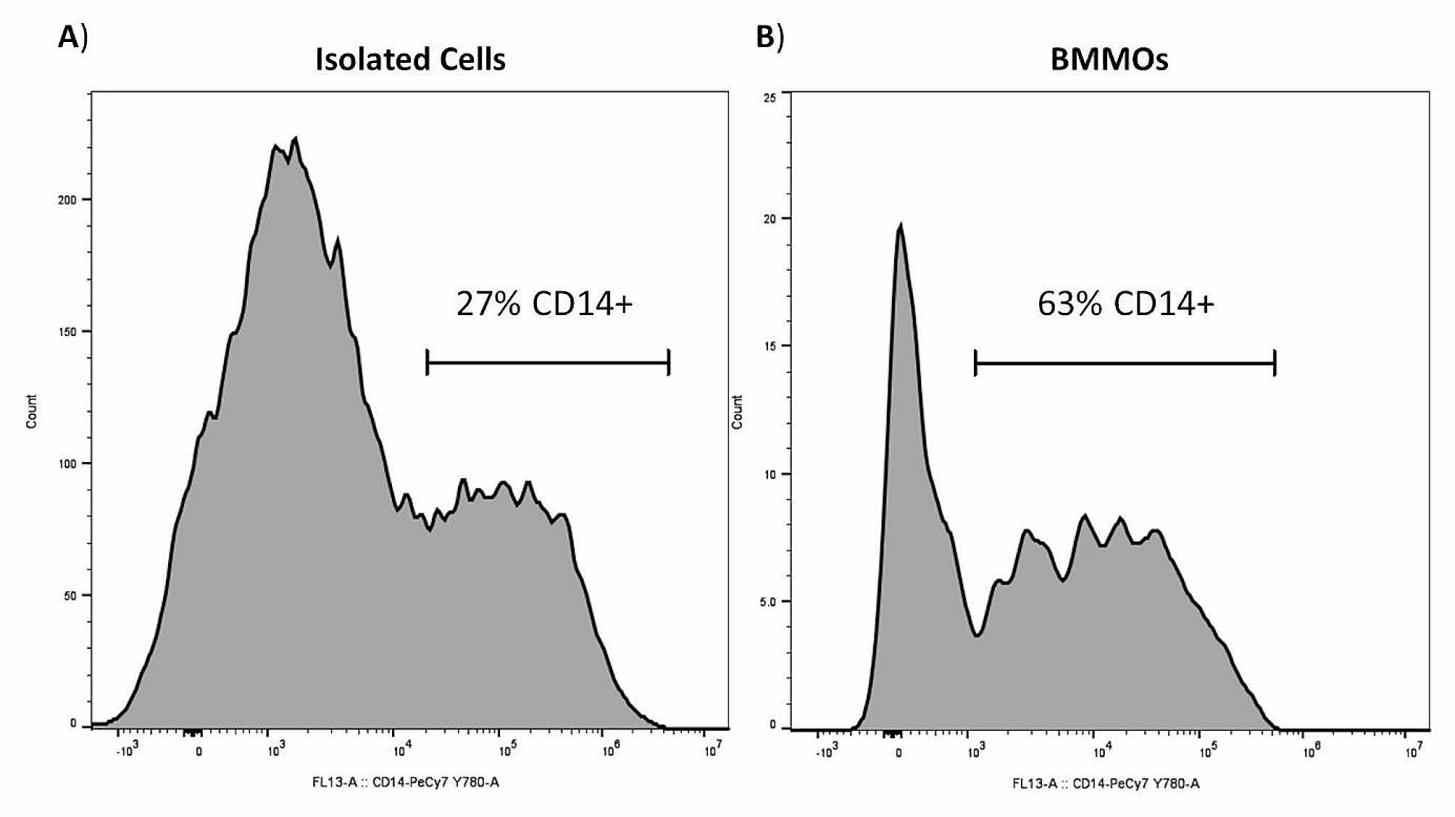
Percentage of CD14 + cells. Flow cytometry analysis was used to determine the percentage of CD14 + cells in the isolated cells (**A**) and bovine mammary macrophage (BMMO) (**B**) populations. The CD14 + population was determined using CD14-PeCy7 antibody and FACS

The percentage of CD14 + cells within the isolated cell population and the BMMO population was determined using flow cytometry analysis. CD14 + cells contributed 27% (Fig. 3A) of the isolated cell population and 63% (Fig. 3B) of the isolated BMMOs.

### Optimal challenge parameters

**Fig. 4:**
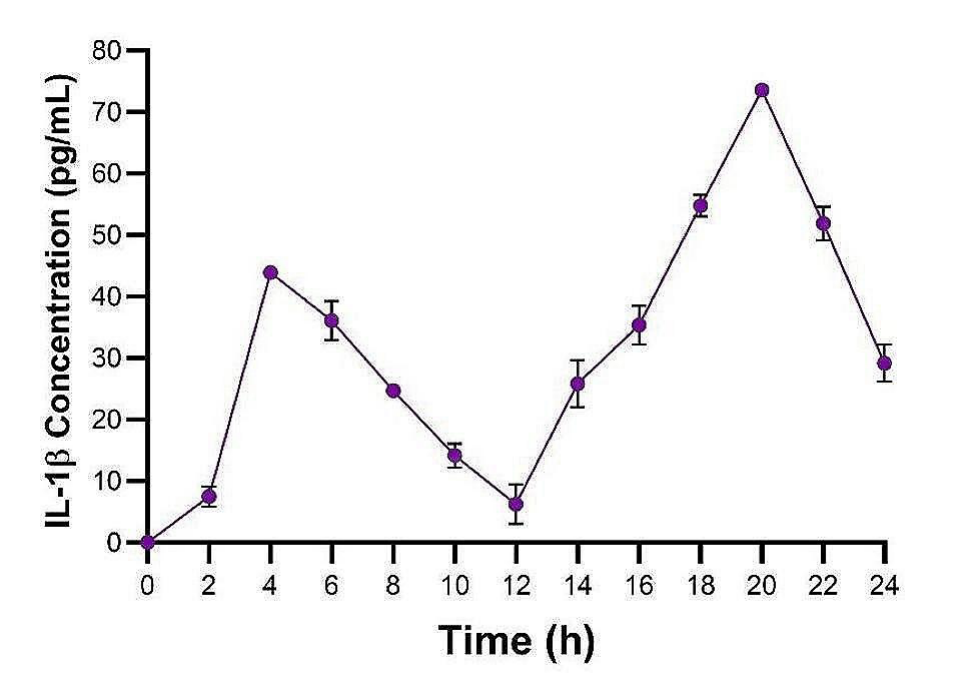
IL-1β produced from BMMOs over 24 h following challenge with *S. uberis* strain 0140J. Bovine mammary macrophages (BMMOs) were isolated from milk and seeded into culture dishes at ~ 50,000 BMMOs/well. BMMOs were challenged with heat-killed *S. uberis* strain 0140J at a multiplicity of infection (MOI) of 50:1 0140 J:BMMO. Supernatants were collected and the concentration of IL-1β was measured by ELISA every 2 h over a 24 h period. Data is presented as mean ± SD with *N* = 6

After establishing the method for isolating BMMOs, the specific parameters to evaluate the macrophage immune response to *S. uberis* needed to be determined to produce consistent and reproducible data. Initially the optimal timepoint to evaluate the immune response was calculated. Isolated BMMOs were challenged with heat-killed *S. uberis* strain 0140J and the IL-1β concentration was measured every 2 h over a 24 h period (Fig. 4). IL-1β production from BMMOs initially increased to ~ 45 pg/mL at 4 h, which steadily decreased to ~ 7 pg/mL at 12 h. IL-1β production from BMMOs then peaked at ~ 75 pg/mL at 20 h followed by another decrease in IL-1β concentration to 24 h. The highest concentration of IL-1β was measured at 20 h.

**Fig. 5:**
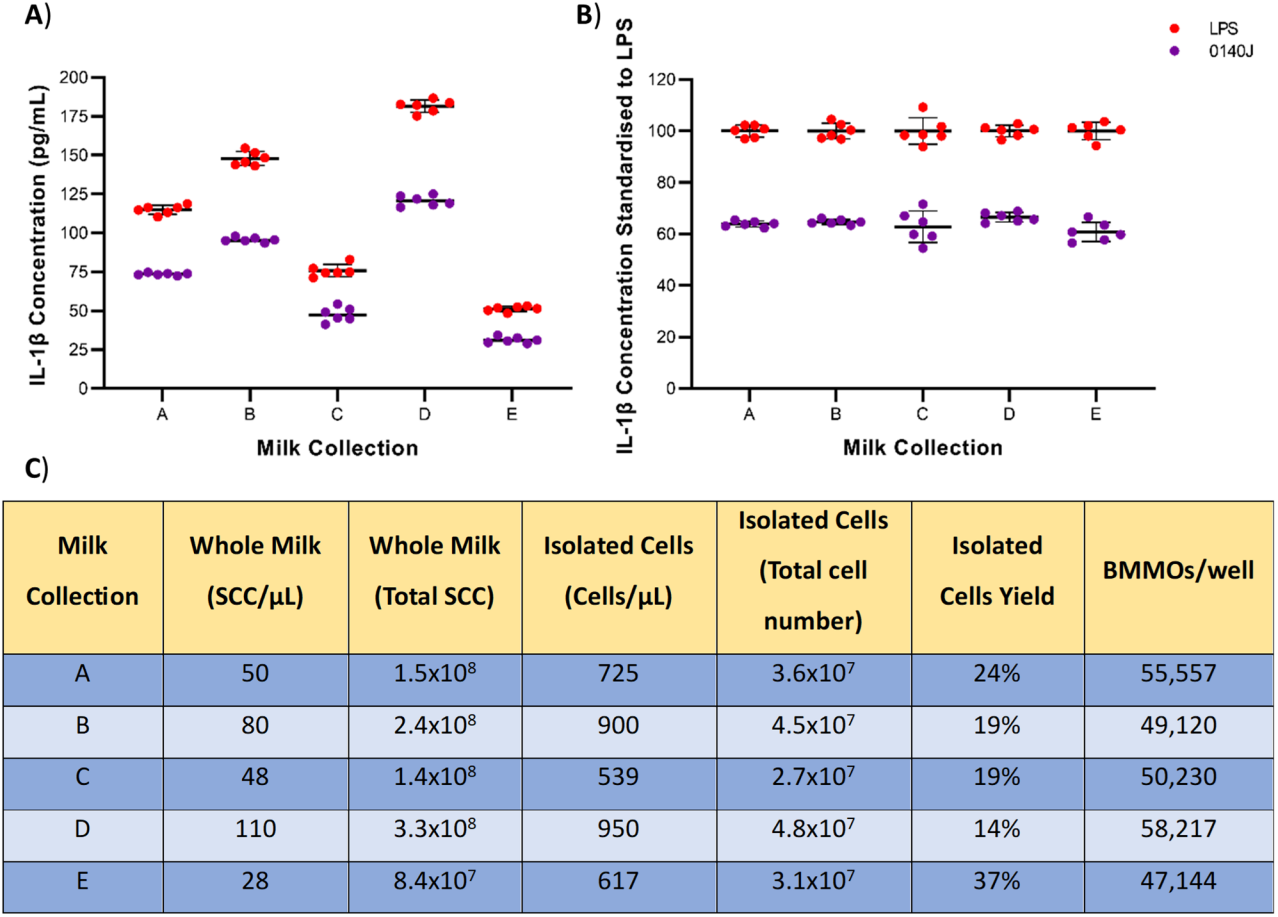
Raw IL-1β concentration vs. IL-1β concentration standardised to LPS. Bovine mammary macrophages (BMMOs) were isolated from milk and seeded into culture dishes at ~ 50,000 BMMOs/well. BMMOs were challenged with heat-killed *S. uberis* strain 0140J at a multiplicity of infection (MOI) of 50:1 0140J:BMMO. Supernatants were collected 20 h after challenge and the concentration of IL-1β was measured by ELISA. BMMOs were stimulated with LPS as a positive control (10 ng/mL). Raw IL-1β concentration was measured from 5 different milk collections (**A**) and standardised to IL-1β produced from BMMOs stimulated with LPS in each corresponding milk collection (**B**). Data is presented as the mean ± SD with *N* = 6. **C**) For each milk collection, the somatic cell count (SCC) per µL was measured using a DeLaval cell counter on whole milk and cells/µL on obtained isolated cells. The total cell numbers were calculated in 3 L milk and isolated cells in 50 mL PBS. The yield was calculated for the percentage of isolated cells obtained from whole milk. Following the BMMO isolation protocol, the numbers of macrophages/well were calculated using a haemocytometer

Stimulated BMMOs from each milk cell preparation were found to produce different concentrations of IL-1β. Comparability between datasets was determined by measuring the IL-1β produced from BMMOs (isolated from five different milk samples) following challenge with *S. uberis* strain 0140J (Fig. 5A) and standardising these values to the corresponding IL-1β concentration following LPS stimulation (Fig. 5B). These data showed that despite the differing concentrations in IL-1β produced from BMMOs stimulated with LPS (51–182 pg/mL) and *S. uberis* strain 0140J (31–121 pg/mL), standardisation resulted in a consistent ratio of IL-1β produced by LPS and *S. uberis* strain 0140J. Challenge of BMMOs with *S. uberis* strain 0140J resulted in an IL-1β concentration at 60–65% of that obtained from LPS stimulation.

**Fig. 6:**
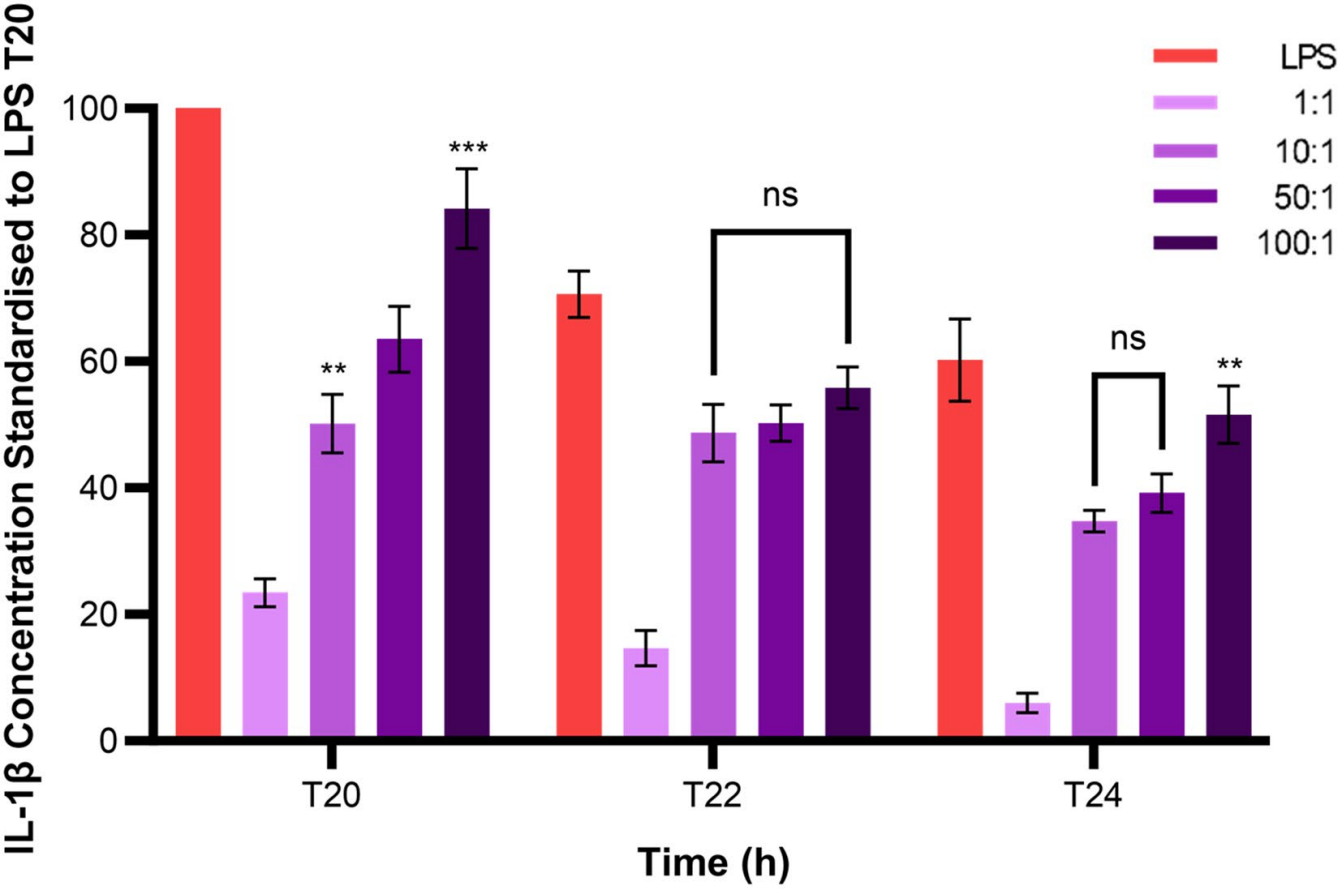
*S. uberis* MOI of BMMOs. Bovine mammary macrophages (BMMOs) were isolated from milk and seeded into culture dishes at ~ 50,000 BMMOs/well. BMMOs were challenged with heat-killed *S. uberis* strain 0140 J at a multiplicity of infection (MOI) of 1:1, 10:1, 50:1 and 100:1 *S. uberis* strain 0140 J:BMMO. Supernatants were collected at 20, 22 and 24 h after challenge and the concentration of IL-1β was measured by ELISA. BMMOs were also unstimulated in a no treatment group and this mean was deducted from the other values, which were then standardised to the LPS positive control (10 ng/mL) at 20 h. Data is presented as mean of this ratio ± SD with *N* = 6. Shapiro-Wilk determined the data to be normally distributed and so was statistically analysed using a two-way ANOVA with Tukey’s multiple comparison post hoc test (***P < 0.001 and **P < 0.01 compared to 50:1 at the corresponding time; ns = not significant)

The optimal multiplicity of infection (MOI) of *S. uberis* strain 0140 J to BMMOs needed to be determined to accurately measure the immune response. Isolated BMMOs were challenged with varying MOIs of heat-killed *S. uberis* strain 0140J at 1:1, 10:1, 50:1 and 100:1. *S. uberis* strain 0140J:BMMO and the IL-1β concentration produced was measured by ELISA 20, 22 and 24 h after challenge (Fig. 6). Results were standardised to the LPS positive control at 20 h. *S. uberis* strain 0140J caused BMMOs to produce 23%, 50%, 65% and 84% IL-1β at 1:1, 10:1, 50:1 and 100:1 respectively at 20 h compared to that obtained from LPS stimulation. The concentration of IL-1β produced from BMMOs 20 h after challenge with *S. uberis* at an MOI of 100:1 was significantly higher than that obtained at an MOI of 50:1 (*P <* 0.0001), which, in turn, was significantly greater than that at an MOI of 10:1 (*P <* 0.001). However, there was no significant differences in the concentration of IL-1β produced from BMMOs 22 h after challenge with *S. uberis* at an MOI of 100:1, 50:1 nor 10:1. At 24 h the concentration of IL-1β produced from BMMOs challenged with *S. uberis* was only significantly greater at an MOI of 100:1 compared to that obtained at 50:1 (*P <* 0.01). Therefore, an MOI of 50:1 *S. uberis* strain 0140 J:BMMO was determined as optimal in measuring IL-1β production from BMMOs 20 h after challenge with *S. uberis*.

**Fig. 7:**
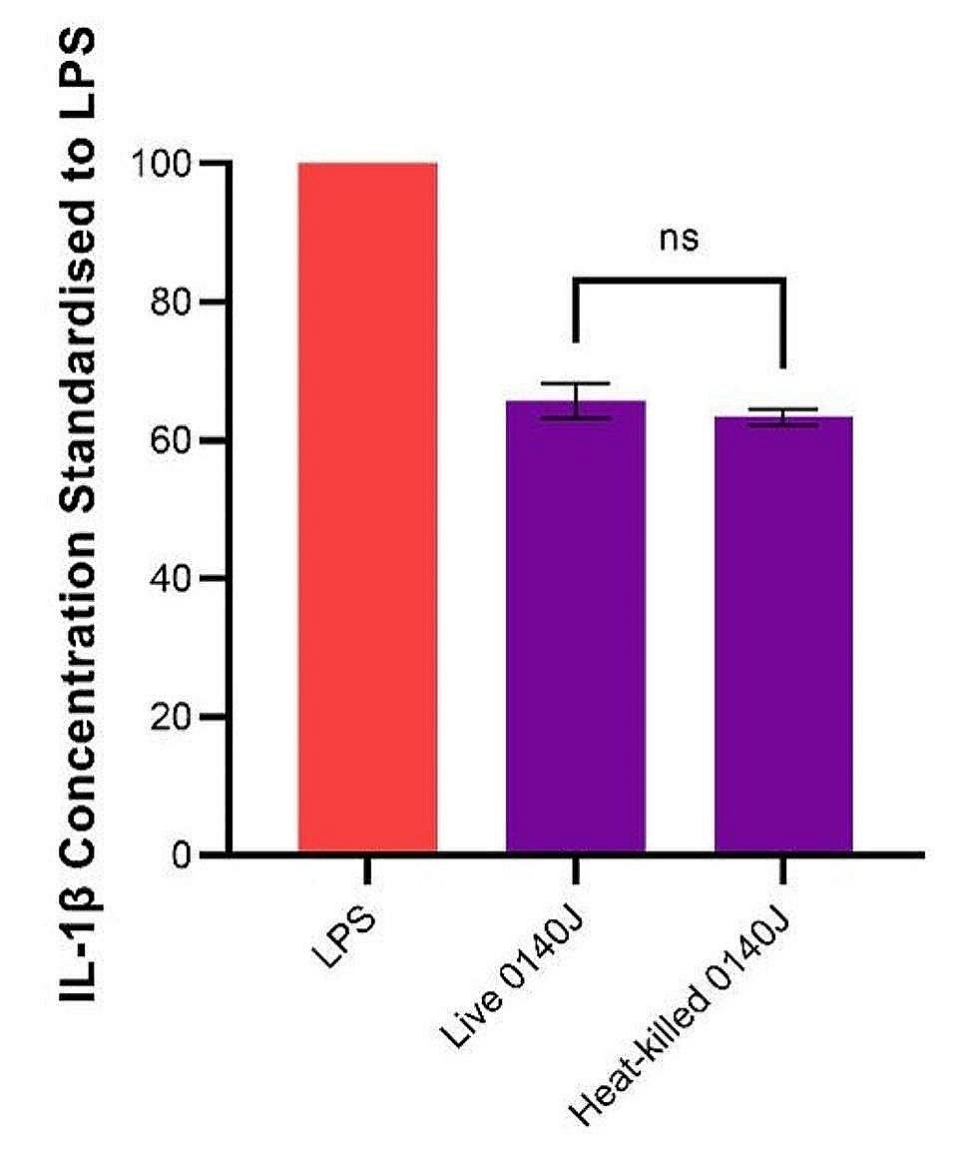
Live vs. heat-killed *S. uberis* strain 0140J. Bovine mammary macrophages (BMMOs) were isolated from milk and seeded into culture dishes at ~ 50,000 BMMOs/well. BMMOs were challenged with live or heat-killed (30 min at 63 °C) *S. uberis* strain 0140J at a multiplicity of infection (MOI) of 50:1 *S. uberis* strain 0140 J:BMMO. Supernatants were collected 4 h after challenge and the concentration of IL-1β was measured by ELISA. BMMOs were also unstimulated in a no treatment group and this mean was deducted from the other values, which were then standardised to the LPS positive control (10 ng/mL). Data is presented as mean ± SD with *N* = 3 (normally assumed) and statistically analysed using an unpaired t-test (ns = not significant)

BMMOs were challenged with either live or heat-killed (30 min at 63 °C) *S. uberis* strain 0140J and the concentration of IL-1β was measured 4 h after challenge. The data showed that there was no statistically significant difference between IL-1β concentration produced by BMMOs stimulated with live or heat-killed *S. uberis* strain 0140J (Fig. 7).

## Discussion

BMMOs are involved in initiating the host response during intramammary infection with *S. uberis* and the ability to investigate their functions ex vivo is required for progression of research on the immunopathogenesis of bovine mastitis. However, no standardised method for isolating and evaluating the BMMO response has been established. Therefore, the aim of this study was to determine a reliable and reproducible method for isolating and measuring the response of BMMOs to bacterial challenge.

A variety of cells have been used experimentally to investigate mastitis pathogenesis. This includes the murine macrophage cell line RAW 246.7 [13], bovine epithelial cell line MAC-T cells [24, 25] and blood derived bovine macrophages [11, 14–17]. Traditionally, macrophages have been classified as M1 or M2 depending on whether they act in a pro- or anti- inflammatory manner. More recent developments in the field depict that this is a restrictive method of classification as macrophages are often completely different depending on their environmental niche and specific cellular properties with modern single-cell profiling techniques demonstrating that M1 and M2 genes are often co-expressed [26]. This underpins the importance of using macrophages from the bovine mammary gland, as they are likely to generate a different response to macrophages differentiated from blood monocytes. This is evident as toll-like receptor 2 (TLR2) was concluded to be involved in the recognition of *S. uberis* when conducted in a murine model [27], which was subsequently shown not to be the case in the context of primary bovine cells [11].

Archer et al., [12] utilised BMMOs from milk, generating an ex vivo model. However, in the present work, isolation of BMMOs using the human CD14 bead kit produced inconsistent results, as the beads appeared no longer cross react with bovine samples, potentially due to a change in antibody used within the kit. Additionally, although some studies isolated BMMOs through FACS, this method was avoided to provide an isolation method for institutions where these facilities are unavailable [18, 22].

The final protocol developed here exploited the fact that macrophages readily stick to plastic while other leukocytes do not [28]. During development of this method, the isolated cells were initially incubated in a tissue culture flask and the non-adherent cells discarded with a view to being able to quantify the BMMOs subsequently released from the plastic to standardise for onward investigations. However, following removal of the BMMOs with ice-cold PBS, these cells showed reduced adherence and activity rendering them less useful for investigation. A similar issue was also reported by Fleit et al., [28]. Consequently, in the final protocol, cells isolated from milk were added directly to culture dishes to immobilise BMMOs and retain their activity in subsequent assays. In accordance with the literature, isolated cells were incubated for varying times (3-18 h) to allow the BMMOs to adhere. Evidence of BMMO adherence was observed after 4 h, however, incubation for 18 h was deemed optimal for BMMO adherence [14–17].

Bovine milk contains a complex cell population. This population includes macrophages, neutrophils and lymphocytes. The proportion of BMMOs within milk varies, estimates within the literature indicate proportions ranging between 10 and 80% of the total cell population in a healthy mammary gland [1, 29]. CD14 is a glycoprotein expressed on monocytes, macrophages, and to a lesser extent neutrophils, and is involved in receptor binding to invading pathogens as part of the innate immune response [30]. The prevalence of CD14 is well established in mouse and human models, however it is not well characterised in BMMOs. FACS analysis found 63% of the adherent cells (BMMOs) were CD14+ (Fig. 3B). CD14 expression has been shown to fluctuate over time, with a proportion of macrophages being CD14 negative [31–34]. Also, removal of BMMOs from culture dishes, even using ice-cold PBS along with the vigorous washing required, may result in damage to the cells, reducing the representation of CD14. Consequently, we can conclude that at least 63% of the immobilised cells (BMMOs) were CD14+.

It was found that BMMOs isolated from different milk samples on different days produced different concentrations of IL-1β following stimulation with *S. uberis* strain 0140J (Fig. 5A). This resulted in issues comparing data between cell preparations/experiments. This did not simply reflect difference in the number of BMMOs immobilised, as a simple relationship between cell number and production of IL-1β was not evident (Fig. 5; sample A produced lower levels of IL-1β than sample B despite a greater number of BMMOs, 55,557/well (sample A) compared to 49,170/well (sample B)). This probably indicated a complex situation relating to both cell number and intrinsically retained activity of the cell population following isolation. Standardisation of activity (in our case production of IL-1β) to our experimental challenge with *S. uberis* strain 0140J with that obtained following stimulation with a standard dose of LPS (Fig. 5B) yielded very consistent data (Fig. 5). This indicated that although each preparation of BMMOs produced different concentrations of IL-1β, the consistent ratio between IL-1β concentration after LPS and *S. uberis* strain 0140J suggested this related to both the quality and quantity of cells used. Removing the sample variation through this standardisation allowed for data to be compared between cell preparations, increasing the utility of the cells prepared/used according to this protocol.

Archer et al., [12] measured the IL-1β concentration 24 h after challenge to evaluate the immune response. To determine the optimal time to focus recording the IL-1β concentration. In this study, two peaks in IL-1β production were observed at 4 and 20 h, with the latter being the greatest (Fig. 4). Macrophages detect pathogens through a variety of methods resulting in downstream signalling pathways that increase pro-IL-1β transcription, and processing via the NLRP3 inflammasome, ultimately resulting in the release of IL-1β in its active, pro-inflammatory form [35, 36]. Macrophages also express cytokine receptors that amplify the macrophage response. Therefore, the binding of secreted IL-1β to IL-1 receptors on neighbouring cells would create a positive feedback loop activating more macrophages resulting in production of further IL-1β. Hence, our interpretation of these data is that the first peak likely represents the initial secretion of IL-1β in response to the pathogen and the second peak is the enhanced response to the pathogen and the initially released IL-1β [36, 37]. Therefore, for the maximal response, IL-1β should be measured at 4 or 20 h post challenge.

As well as the optimal time to measure the BMMO immune response to *S. uberis*, the optimal MOI was determined (Fig. 6). An MOI of 50:1 was selected as optimal as this challenge dose produced a reproducible response of 60–65% of that obtained with a standard dose of LPS. This allowed for reliable measurement of both greater and lower production of IL-1β within the range of the positive control condition (challenge with LPS) during the subsequent investigation. Challenge with MOIs 10:1, 50:1 and 100:1 resulted in significantly different levels of IL-1β at 20 h after challenge, further indicating that the measurement of IL-1β concentration using an MOI of 50:1 at 20 h post challenge was optimal to investigate changes (higher and lower) in the production of IL-1β.

To accurately control the *S. uberis* strain 0140J:BMMO MOI throughout the 20 h after challenge, IL-1β concentration was measured following challenge with either live or heat-killed *S. uberis* strain 0140J. No significant difference was found, implying that this reaction was not dependent on or enhanced by factors secreted by live bacterial cells. Günther et al., [11] also reported no difference between live or heat-killed *S. uberis* in inducing expression of immune genes in primary bovine mammary epithelial cells. Use of killed bacterial challenge, removes the variability of bacterial number that may occur due to bacterial growth and permitted experiments to be conducted in the presence of antimicrobials allowing comparisons between datasets and analysis of the BMMO immune response.

## Conclusion

A method based on cell isolation and adherence to plastic was established to isolate a yield of approximately 1.8 × 10^6^ BMMOs from 3 L of raw bovine milk. Seeding of approximately 50,000 BMMOs/well into each compartment of a 24-well culture dish produced reliable and reproducible data with regard to production of IL-1β in response to challenge with *S. uberis* compared to a standard dose of LPS. This standardised approach will allow for further research to be conducted regarding the BMMO response to intramammary pathogens, utilising macrophages specifically differentiated in the mammary gland niche.

## Methods

### Isolation of BMMOs from milk

Raw milk was collected from bulk tank at the University of Nottingham, Sutton Bonington campus dairy centre. The somatic cell count (SCC) was determined using a DeLaval Cell Counter (DCC). Intramammary infection is often accompanied by an elevation in the SCC. Consequently, milk with a SCC > 200 cells/µL was discarded. Milk with a SCC of < 200 cells/µL was processed to obtain the milk cell population. Equal volumes of milk and PAE buffer (PBS + 10% acid-citrate dextrose (citric acid, Sigma, 33,114; tris-sodium citrate, Fisher Chemical, S/P500/53; D-(+)-glucose, Sigma, G7528) + 20 mM EDTA (Thermo Scientific, J15694-AP) were centrifuged (40 min, 600xg) at 15 °C, with acceleration 9 and deceleration 1 (Thermo Scientific, Megafuge™ 16R). The pellet was resuspended and washed with PBS and cells obtained by centrifugation (10 min, 300xg) at 15 °C, with acceleration 9 and deceleration 5. Cells were then washed twice in PBS with the addition of 2% antibiotic-antimycotic (Sigma, A5955) and 0.5 µg/mL Amphotericin B (Gibco, 15,290,018) and cell density was measured (DCC). Finally, cells were resuspended in IMDM (Iscove’s modified Dulbecco’s medium) containing L-glutamate and 25 mM HEPES (Gibco, 12440-046) + 10% foetal bovine serum (FBS, Sigma, F7524) + 2% antibiotic-antimycotic + 0.5 µg/mL Amphotericin B and plated at a density of 1 × 10^6^/well in a Nunc™ 24-well cell culture treated plate (Thermo Scientific, 142,475) and incubated overnight (18 h) at 37 °C and 5% CO_2_.

Media was removed and each well was washed with PBS heated to 37 °C to remove any nonadherent cells, leaving only the adhered BMMOs. Three wells were set aside for each plate where, following disposal of nonadherent cells, BMMOs from these wells were removed using ice cold PBS and the cell number calculated using a haemocytometer (Marienfeld, 0640030) to verify the number of BMMOs in each well (~ 50,000). Cell viability was assessed by exclusion using 0.4% trypan blue solution (Sigma, T814) (92–94%) and consistent macrophage morphology was observed.

### Flow cytometry

A sample of isolated somatic cells and BMMOs were collected and washed three times by centrifugation at 300xg for 5 min and resuspended in FACS buffer (0.5% Bovine Serum Albumin (BSA, Fisher Scientific, BP1605) in PBS). 1 µg PE-Cy7 mouse anti-human CD14 (BD Pharmingen, 560,919, clone M5E2) was incubated with 100 µL of each sample for 1 h at room temperature in the dark. Washing was repeated followed by incubation in 4% paraformaldehyde (Sigma, 100,496) for 15 min at room temperature. Samples were centrifuged and resuspended in ice cold PBS and stored at 4 °C in the dark until analysed on a CytoFLEX S flow cytometer (Beckman Coulter).

### Bacterial culturing conditions

*S. uberis* strain 0140J (strain ATCC BAA-854/0140J), originally isolated from a clinical case of bovine mastitis in the UK, was cultured in Brain Heart Infusion (BHI) media (Oxoid, CM1135) at 37 °C overnight. Bacterial cultures were washed 3 times in PBS at 5,000xg for 3 min and then heat-killed at 63 °C for 30 min. Cultures were resuspended in IMDM at an optical density (OD) of 1 at 600 nm wavelength.

### BMMO challenge

Following isolation, BMMOs were challenged with either live or heat-killed *S. uberis* strain 0140J at a multiplicity of infection (MOI) of 1:1, 10:1, 50:1 or 100:1 bacterium:BMMO and lipopolysaccharide (LPS) (10 ng/mL; isolated from *E. coli* 0111:B4, Millipore, LPS25) as a positive control and IL-1β measured up to 24 h after challenge.

### ELISA

Bovine IL-1β was detected by ELISA using the Invitrogen Reagent Kit (ESS0027) following manufacturer’s instructions. Coating antibody was diluted in BupH Carbonate/Bicarbonate Buffer (0.2 M, Invitrogen, 28,382) and incubated overnight at room temperature in 96-well plates (Thermo Scientific, clear flat-bottom immune nonsterile, 3355). Plates were aspirated and incubated for 1 h at room temperature in blocking buffer (4% BSA and 5% sucrose (Sigma, S0389) in PBS). Wells were washed with PBS + 0.05% Tween-20 (Sigma, P1379) and the detection antibody and streptavidin-HRP (horseradish peroxidase) were diluted in reagent diluent (4% BSA in PBS).

Absorbance was measured at wavelengths of 450 and 550 nm using a Varioskan® Flash multimode plate reader (Thermo Scientific). Optical imperfections were corrected for by subtracting the 550 nm reading from the 450 nm. A standard curve was generated and sample IL-1β concentrations were interpolated.

### Statistical analysis

Data were analysed using GraphPad Prism 10. To determine if data were normally distributed a Shapiro-Wilk test was used. Normally distributed data were statistically analysed using either a 2-way ANOVA with Tukey’s multiple comparisons post hoc test or an unpaired t-test. When data were found not to be normally distributed, a Kruskal-Wallis with Dunn’s multiple comparisons post hoc test was used to determine significance. A value of *P* ≤ 0.05 was considered to indicate a statistically significant difference. Spearman rank-order correlation coefficient was used to determine association, with nonlinear regression to calculate coefficient of determination. Flow cytometry data were analysed using FlowJo v10.9.0.

### Electronic supplementary material

Below is the link to the electronic supplementary material.

Supplementary Material 1

Supplementary Material 2

Supplementary Material 3

Supplementary Material 4

Supplementary Material 5

## Data Availability

No datasets were generated or analysed during the current study.

## Abbreviations

BMMO: bovine mammary macrophage
CD14: cluster of differentiation 14
DCC: DeLaval cell counter
ELISA: enzyme-linked immunosorbent assay
FACS: fluorescence activated cell sorting
IL-1β: interleukin-1 beta
LPS: lipopolysaccharide
MOI: multiplicity of infection
NF-κB: nuclear factor kappa B
PBMC: peripheral blood mononuclear cells
SCC: somatic cell count

## References

[1] Paape M, Mehrzad J, Zhao X, Detilleux J, Burvenich C (2002). Defense of the bovine mammary gland by polymorphonuclear neutrophil leukocytes. J Mammary Gland Biol Neoplasia.

[2] Rainard P, Foucras G, Boichard D, Rupp R (2018). Invited review: low milk somatic cells count and susceptibility to mastitis. J Dairy Sci.

[3] Rainard P, Gilbert FB, Germon P (2022). Immune defenses of the mammary gland epithelium of dairy ruminants. Front Immunol.

[4] Grant RG, Finch JM (1997). Phagocytosis of *Streptococcus uberis* by bovine mammary macrophages. Res Vet Sci.

[5] Pidwill GR, Gibson JF, Cole J, Renshaw SA, Foster SJ (2020). The role of macrophages in *Staphylococcus aureus* infection. Front Immunol.

[6] Watts JL (1988). Etiological agents of bovine mastitis. Vet Microbiol.

[7] Klaas IC, Zadoks RN (2018). An update on environmental mastitis: challenging perceptions. Transbound Emerg Dis.

[8] Bradley AJ, Leach KA, Breen JE, Green LE, Green MJ (2007). Survey of the incidence and aetiology of mastitis on dairy farms in England and Wales. Vet Rec.

[9] UK-VARSS. 2019. UK Veterinary Antibiotic Resistance and Sales.

[10] Moyes K, Drackley JK, Morin DE, Rodriguez-Zas SL, Everts R, Lewin HA (2010). Mammary gene expression profiles during an inflammatory challenge reveal potential mechanisms linking negative energy balance with impaired immune response. Physiol Genomics.

[11] Günther J, Czabanska A, Bauer I, Leigh JA, Holst O, Seyfert HM. *Streptococcus uberis* strains isolated from the bovine mammary gland evade immune recognition by mammary epithelial cells, but not of macrophages. Vet Res. 2016;47(13). 10.1186/s13567-015-0287-8 PMC4704416 26738804

[12] Archer N, Egan SA, Coffey TJ, Emes RD, Addis MF, Ward PN (2020). A paradox in bacterial pathogenesis: activation of the local macrophage inflammasome is required for virulence of *Streptococcus uberis*. Pathogens.

[13] Ascanius SR, Hansen MS, Ostenfeld MS, Rasmussen JT (2021). Milk-derived extracellular vesicles supress inflammatory cytokine expression and nuclear factor-κB activation in lipopolysaccharide-stimulate macrophages. Dairy.

[14] Burr S, Thomas C, Brownlie J, Offord V, Coffey TJ, Werling D (2012). Potential evidence for biotype-specific chemokine profile following BVDV infection of bovine macrophages. Vet Immunol Immunopathol.

[15] Menge C, Loos D, Bridger PS, Barth S, Werling D, Baljer G (2015). Bovine macrophages sense *Escherichia coli* Shiga toxin 1. Innate Immun.

[16] Gibson AJ, Woodman S, Pennelegion C, Patterson R, Stuart E, Hosker N (2016). Differential macrophage function in Brown Swiss and Holstein Friesian cattle. Vet Immunol Immunopathol.

[17] Jensen K, Gallagher IJ, Kaliszewska A, Zhang C, Abejide O, Gallagher MP (2016). Live and inactivated *Salmonella enterica* serovar typhimurium stimulates similar but distinct transcriptome profiles in bovine macrophages and dendritic cells. Vet Res.

[18] Hébert A, Sayasith K, Sénéchal S, Dubreuil P, Lagacé J (2000). Demonstration of intracellular *Staphylococcus aureus* in bovine mastitis alveolar cells and macrophages isolated from naturally infected cow milk. FEMS Micro Lett.

[19] Hussen J, Schuberth HJ (2017). Heterogeneity of bovine peripheral blood monocytes. Front Immunol.

[20] Garcia-Sánchez M, Jiménez-Pelayo L, Horcajo P, Regidor-Cerrillo J, Ólafsson EB, Bhandage AK et al. Differential response of bovine monocyte-derived macrophages to infection by Neospora Caninum isolated of high and low virulence. Front Immunol. 2019;10(915). 10.3389/fimmu.2019.00915 PMC6503000 31114577

[21] Ladero-Auñon I, Molina E, Holder A, Kolakowski J, Harris H, Urkitza A (2021). Bovine neutrophils release extracellular traps and cooperate with macrophages in *Mycobacterium avium* subsp. paratuberculosis. Clearance in vitro. Front Immunol.

[22] Silva VM, Souza MT, Blagitz MG, Souza FN, Batista CF, Alves AJ (2021). Milk lymphocyte profile and macrophages functions: new insights into the immunity of the mammary gland in quarters infected with *Corynebacterium bovis*. BMC Vet Res.

[23] Sandgren CH, Nordling K (1991). Björk I. isolation and phagocytic properties of neutrophils and other phagocytes form nonmastitic bovine milk. J Dairy Sci.

[24] Prado ME, Almeida RA, Ozen C, Luther DA, Lewis MJ, Headrick SJ, Oliver SP (2011). Vaccination of dairy cows with recombinant Streptococcus uberis adhesion molecule induces antibodies that reduce adherence to and internalization of S. uberis into bovine mammary epithelial cells. Vet Immunol Immunopathol.

[25] Wang X, Liu M, Geng N, Du Y, Li Z, Gao X (2022). Staphylococcus aureus mediates pyroptosis in bovine mammary epithelial cell via activation of NLRP3 inflammasome. Vet Res.

[26] Becker D, Weikard R, Hadlich F, Kühn C. Single-cell RNA sequencing of freshly isolated bovine milk cells and cultured primary mammary epithelial cells. Sci Data. 2021;8(177). 10.1038/s41597-021-00972-1 PMC8282601 34267220

[27] Wan Z, Wang X, Liu M, Zuo J, Xu Y, Han X et al. Role of toll-like receptor 2 against *Streptococcus uberis* infection in primary mouse mammary epithelial cells. Intern Immunopharmacol. 2020;79. 10.1016/j.intimp.2019.106142 31931293

[28] Fleit SA, Fleit HB, Zolla-Pazner S (1984). Culture and recovery of macrophages and cell lines from tissue culture-treated and -untreated plastic dishes. J Immunol Methods.

[29] Alnakip ME, Quintela-Baluja M, Böhme K, Fernández-No I, Caamaño-Antelo S, Calo-Mata P, Barros-Velázques J. The immunology of mammary gland of dairy ruminants between healthy and inflammatory conditions. J Vet Med. 2014. 10.1155/2014/659801 PMC4590879 26464939

[30] Antal-Szalmas P, Strijp JA, Weersink AJ, Verhoef J, Van Kessel KP (1997). Quantitation of surface CD14 on human monocytes and neutrophils. J Leukoc Biol.

[31] Sladek Z, Rysanek D (2008). Expression of macrophage CD14 receptor in the course of experimental inflammatory responses induced by lipopolysaccharide and muramyl dipeptide. Vet Med.

[32] Sladek Z, Rysanek D (2014). CD14 expression, apoptosis and necrosis in resident and inflammatory macrophages from virgin bovine mammary gland. Vet Med.

[33] Lambert C, Preijers FWMB, Yanikkaya Demirel G, Sack U (2017). Monocytes and macrophages in flow: an ESCCA initiative on advanced analyses of monocyte lineage using flow cytometry. Cytometry B Clin Cytom.

[34] Lai TY, Cao J, Ou-Yang P, Tsai CY, Lin CW, Chen CC et al. Difference methods of detaching adherent cells and their effects on the cell surface expression of Fas receptor and Fas ligand. Sci Rep. 2022;12(5713). 10.1038/s41598-022-09605-y PMC8983651 35383242

[35] Chen N, Xia P, Li S, Zhang T, Wang TT, Zhu J (2017). RNA sensors of the innate immune system and their detection of pathogens. IUBMB Life.

[36] Kaneko N, Kurata M, Yamamoto T, Morikawa S, Masumoto J. The role of interleukin-1 in general pathology. Inflamm Regener. 2019;39(12). 10.1186/s41232-019-0101-5 PMC6551897 31182982

[37] Ley K, Pramod AB, Croft M, Ravichandran KS, Ting JP (2016). How mouse macrophages sense what is going on. Front Immunol.

