# Supplementary material for: Reproducible isolation of bovine mammary macrophages for analysis of host pathogen interactions
Source: BMC Vet Res. 2024 Mar 9;20:96. doi: 10.1186/s12917-024-03944-w (PMC10924389; doi:10.1186/s12917-024-03944-w)
Supplement: Supplementary file 1 — Supplementary Material 1 [file 12917_2024_3944_MOESM1_ESM.pdf]

Supplemental Data 1. Cell isolation numbers and yield. For each 3L of milk collected, the whole milk somatic cell count (SCC/µL) was measured using a DeLaval cell counter and total cell number calculated. During the cell isolation protocol, the cells were resuspended in 50 mL PBS and cells/µL were measured and total cell number calculated. The yield was calculated for the percentage of isolated cells obtained from whole milk. Following washing of cells to remove non-adherent cells, from the 3 wells set aside, bovine mammary macrophages (BMMOs) were removed and the number of BMMOs/well was calculated using a haemocytometer. The mean for each column is presented in the bottom row (N=96).

Whole Milk (SCC/µL)	Whole Milk (Total SCC)	Isolated Cells (cells/µL)	Isolated Cells (Total cell number)	Isolated Cell Yield (%)	BMMOs/well
36	$1.08x10^8$	880	$4.40x10^7$	40.7	55,911
36	$1.08x10^8$	892	$4.46x10^7$	41.3	68,842
51	$1.53x10^8$	810	$4.05x10^7$	26.5	51,363
116	$3.48x10^8$	790	$3.95x10^7$	11.4	65,233
90	$2.70x10^8$	600	$3.00x10^7$	11.1	48,487
100	$3.00x10^8$	740	$3.70x10^7$	12.3	59,203
73	$2.19x10^8$	1290	$6.45x10^7$	29.5	57,147
129	$3.87x10^8$	651	$3.26x10^7$	8.4	41,498
70	$2.10x10^8$	890	$4.45x10^7$	21.2	57,440
100	$3.00x10^8$	900	$4.50x10^7$	15.0	62,581
50	$1.50x10^8$	650	$3.25x10^7$	21.7	56,480
150	$4.50x10^8$	940	$4.70x10^7$	10.4	39,423
110	$3.30x10^8$	720	$3.60x10^7$	10.9	65,815
90	$2.70x10^8$	690	$3.45x10^7$	12.8	67,298
160	$4.80x10^8$	460	$2.30x10^7$	47.9	60,297
100	$3.00x10^8$	840	$4.20x10^7$	14.0	63,639
80	$2.40x10^8$	820	$4.10x10^7$	17.1	61,391
40	$1.20x10^8$	300	$1.50x10^7$	12.5	55,414
70	$2.10x10^8$	590	$2.95x10^7$	14.0	58,072
86	$2.58x10^8$	700	$3.50x10^7$	13.6	52,098
86	$2.58x10^8$	840	$4.20x10^7$	16.3	52,686
60	$1.80x10^8$	700	$3.50x10^7$	19.4	44,533
75	$2.25x10^8$	1100	$5.50x10^7$	24.4	60,277
80	$2.40x10^8$	600	$3.00x10^7$	12.5	68,741
150	$4.50x10^8$	660	$3.30x10^7$	7.3	60,050
50	$1.50x10^8$	725	$3.63x10^7$	24.2	55,557
60	$1.80x10^8$	540	$2.70x10^7$	15.0	62,276
45	$1.35x10^8$	650	$3.25x10^7$	24.1	44,736
35	$1.05x10^8$	400	$2.00x10^7$	19.0	44,093
48	$1.44x10^8$	630	$3.15x10^7$	21.9	42,615
40	$1.20x10^8$	555	$2.78x10^7$	23.1	56,035
70	$2.10x10^8$	690	$3.45x10^7$	16.4	62,141
97	$2.91x10^8$	1468	$7.34x10^7$	25.2	62,592
110	$3.30x10^8$	540	$2.70x10^7$	8.2	51,009
52	$1.56x10^8$	450	$2.25x10^7$	14.4	57,823
196	$5.88x10^8$	768	$3.84x10^7$	6.5	58,028
48	$1.44x10^8$	464	$2.32x10^7$	16.1	47,244
86	$2.58x10^8$	660	$3.00x10^7$	11.6	56,747
69	$2.07x10^8$	900	$4.50x10^7$	21.7	45,034
45	$1.38x10^8$	550	$2.75x10^7$	19.9	66,417
162	$4.86x10^8$	997	$4.99x10^7$	10.3	53,599
36	$1.08x10^8$	513	$2.57x10^7$	23.8	68,386
110	$3.30x10^8$	1000	$5.00x10^7$	15.2	68,328
55	$1.65x10^8$	575	$2.88x10^7$	17.5	50,612

70	2.10×10^8	540	2.70×10^7	12.9	58,891
40	1.20×10^8	600	3.00×10^7	25.0	44,652
57	1.71×10^8	578	2.89×10^7	16.9	41,620
135	4.05×10^8	850	4.25×10^7	10.5	61,495
64	1.92×10^8	650	3.25×10^7	16.9	46,471
76	2.28×10^8	800	4.00×10^7	17.5	53,347
140	4.20×10^8	640	3.20×10^7	7.6	56,145
80	2.40×10^8	900	4.50×10^7	18.8	49,120
140	4.20×10^8	800	4.00×10^7	9.5	60,043
44	1.32×10^8	468	2.34×10^7	17.7	46,414
42	1.26×10^8	694	3.47×10^7	27.5	45,267
58	1.74×10^8	688	3.44×10^7	19.8	50,174
64	1.92×10^8	1360	6.80×10^7	35.4	40,135
36	1.08×10^8	611	3.06×10^7	28.3	46,867
36	1.08×10^8	487	2.44×10^7	22.6	41,635
141	4.23×10^8	1000	5.00×10^7	11.8	59,209
140	4.20×10^8	1059	5.30×10^7	12.6	63,322
72	2.16×10^8	950	4.75×10^7	22.0	50,520
23	6.90×10^7	430	2.15×10^7	31.2	40,670
57	1.71×10^8	1200	6.00×10^7	35.1	51,985
40	1.20×10^8	450	2.25×10^7	18.8	52,929
63	1.89×10^8	783	3.92×10^7	20.7	64,172
50	1.50×10^8	422	2.11×10^7	14.1	40,060
65	1.95×10^8	388	1.94×10^7	9.9	44,172
53	1.59×10^8	849	4.25×10^7	26.7	56,440
48	1.44×10^8	539	2.70×10^7	18.8	50,230
58	1.74×10^8	342	1.71×10^7	9.8	48,823
78	2.34×10^8	577	2.89×10^7	12.4	53,850
110	3.30×10^8	1125	5.63×10^7	17.1	60,686
121	3.63×10^8	1305	6.53×10^7	18.0	62,421
50	1.50×10^8	565	2.83×10^7	18.9	50,022
84	2.52×10^8	650	3.25×10^7	12.9	56,179
110	3.30×10^8	950	4.75×10^7	14.4	58,217
65	1.95×10^8	785	3.93×10^7	20.2	52,390
47	1.41×10^8	679	3.40×10^7	24.1	47,645
44	1.32×10^8	426	2.13×10^7	16.1	47,737
90	2.70×10^8	1044	5.22×10^7	19.3	61,568
123	3.69×10^8	1422	7.11×10^7	19.3	58,175
125	3.75×10^8	1687	8.44×10^7	22.5	59,399
68	2.04×10^8	964	4.82×10^7	23.6	52,275
83	2.49×10^8	750	3.75×10^7	15.1	50,655
67	2.01×10^8	852	4.26×10^7	21.2	46,326
65	1.95×10^8	741	3.71×10^7	19.0	46,905
65	1.95×10^8	763	3.82×10^7	19.6	53,143
60	1.80×10^8	656	3.28×10^7	18.2	49,464
50	1.50×10^8	1030	5.15×10^7	34.3	65,534
65	1.95×10^8	728	3.64×10^7	18.7	53,989
54	1.62×10^8	518	2.59×10^7	16.0	40,814
70	2.10×10^8	1600	8.00×10^7	38.1	67,455
28	8.40×10^7	617	3.09×10^7	36.8	47,144
43	1.29×10^8	579	2.90×10^7	22.5	44,556
61	1.83×10^8	516	2.58×10^7	14.1	49,133
**76**	**2.29×10^8**	**758**	**3.79×10^7**	**19.1**	**54,041**
